# The Collapse of Matteism

**Published:** 1892-08-20

**Authors:** 


### THE DOCTOR'S ARMCHAIR.

### THE COLLAPSE OF MATTEISM.

THE *British Medical Journal* published last week the full report of the Committee, which had undertaken the investigation of Count Mattei's so-called "Cancer cure." Cancer is one of those diseases which have baffled the practitioners of the medical art in every country. The best physicians and surgeons sadly acknowledge that they have not hitherto been able to discover or devise any substance or method which could with propriety be called a veritable "cure" for cancer. It is, however, a popular error to suppose that nothing effectual can be done for any kind of growth which is really cancerous in its nature. The actual fact is that many tumours, which, if left alone, would ultimately prove themselves to be cancerous, are removed by judicious surgeons at a very early stage of their development, and never recur; in other words, a real cure of cancer is effected in a very large number of cases of this kind.

It still remains true, however, that cancerous growths which have attained to any considerable development almost invariably compass the death of the patient sooner or later. Under these circumstances, it is not at all surprising that all kinds of rational and irrational methods of treatment should be resorted to by practitioners, or that despairing patients should desert orthodox professors of medicine, who tell them the truth, for fairspoken outsiders who promise them relief and cure. No reasonable and kind-hearted doctor can blame severely a patient who has been avowedly "given up" by orthodoxy for trying heterodoxy. It is not in human nature for the patient to refrain from venturing upon the methods of those who promise so fair, and the doctor himself, knowing how he would feel if he were in his patient's place, puts a humane embargo on his otherwise protesting tongue.

But the difference between orthodox and heterodox medicine is this, that the orthodox practitioner, when he finds he cannot cure, has the candour to say so; whilst the outsider, less scrupulous, and certainly much less successful, continues to pile promise upon promise until the patient drops into the grave. If we ask that the same stringent rules should be applied to heterodox as to orthodox practice, the most prejudiced anti-faculty reader must admit that we are at least fair. Now it is a standing rule with right-minded medical men that a method of treatment which constantly fails in practice shall be given up. This rule is so obviously reasonable that it would be childish to defend it. The same rule, we submit, ought to be applied to heterodox practice. If heterodox practitioners were as candid and straightforward as they should be, they would apply it for themselves. But if such practitioners will not apply such a rule for themselves, and that on the sole ground of a diminution of profits, surely a public that has any discrimination at all ought to have sense enough to apply it in its own defence.

Now, an impartial Medical Committee, consisting of five competent and trusted practitioners, a Committee approved by representative Matteists themselves, has declared in the pages of the *British Medical Journal* and in those of the *Review of Reviews*, that in its deliberate judgment the Mattei remedies have no effect upon cancer whatsoever, any more than plain water has. It is to be remembered that the Committee had five selected cases of cancer under its observation for a whole year. Those cases were selected and approved by the Matteists themselves; they were placed under the entire and absolute control of the cancer-curing Count's representatives in every particular, no other person being permitted to influence their treatment in a single detail. Yet the Committee and their Registrar have united in affirming that every single case was worse at the end than at the beginning of the year; some a little worse, and others a great deal.

It is plain, then, that not only is Matteism not a cure for cancer, but that it has no effect upon cancer whatsoever. This result was, of course, anticipated by intelligent medical men, because Matteism has been tried by a good many orthodox practitioners, and has been found in every instance to fail. The public should, we think, accept this verdict as final; and efforts should be made to put a stop to a system of quackery which is both misleading and dangerous.

The old parrot cry that doctors would rather see patients die than try any unorthodox method of treatment has been amply disproved by the Mattei investigation. Not only were there found five orthodox practitioners who were willing to be convinced if facts should be forthcoming, but many others, equally orthodox and equally trustworthy, expressed their readiness to take part in the investigation if necessity should arise. The truth is that of all people who desire to find cures for diseases medical men are by far the most anxious. Nothing can be more obvious than this. The reputation, the life and soul, the bread and butter of the medical profession depends upon its power to cure, and upon nothing else. The very same motives which impel the farmer to cultivate his ground and to reap his crops, the lawyer to strain every nerve to win his case, and the man of business to be first in the morning at his office, and last in the evening, impel the medical man to put forth every possible effort which an educated intelligence can suggest for the comfort and cure of his patient.

The exposure of Matteism should be the exposure and extinction of every other form of medical quackery. It is not true that medical men, as a class, are unwilling to try new and unrecognised remedies. The very opposite is the truth. There are in this country hundreds, nay thousands, of medical men of the highest degree of competency, who devote a great portion of their time to this very business of finding out and testing new remedies. Anything whatever which suggests the least hope of a cure they are willing to experiment with. All this is now proved beyond a doubt; and if the public were wise, it would insist that no remedy of any kind should be employed upon sick persons, or even sold over the counter, until it had been approved by a competent medical tribunal. Whatever may have been the case in past times, the medicine of to-day is thoroughly scientific and honest. The public may safely trust the profession, which is rapidly purging itself even of its harmless prejudices. Quackery, both licensed and unlicensed, ought certainly now to die.

